# Postdictive confidence (but not predictive confidence) predicts eyewitness memory accuracy

**DOI:** 10.1186/s41235-018-0125-4

**Published:** 2018-08-29

**Authors:** Thao B. Nguyen, Erica Abed, Kathy Pezdek

**Affiliations:** Department of Psychology, Claremont Graduate University, Claremont, CA 91711 USA

**Keywords:** Judgments of learning, Confidence-accuracy, Metamemory, Cross-race effect, Eyewitness memory

## Abstract

If testing conditions are uncontaminated, confidence at test reliably predicts eyewitness memory accuracy. Unfortunately, information about eyewitness postdictive confidence (at the time of the identification test) is frequently unavailable or not well documented. In cases where postdictive confidence is unavailable, a useful indicator of eyewitness accuracy might be an eyewitness’s predictive confidence made shortly after the event. How do the accuracy of predictive and postdictive confidence judgments compare; and do variables reported to affect memory (e.g. exposure duration, face race) affect the reliability of the confidence-accuracy relationship for predictive and postdictive judgments? In two experiments, we tested the accuracy of memory predictions (immediate and delayed judgments of learning [JOLs]) and postdictions (confidence) for same- and cross-race faces. Although delayed high JOLs were indicative of higher recognition memory accuracy than delayed low JOLs for both same- and cross-race faces, the accuracy of even high predictive JOLs was objectively low. Postdictive confidence was a far stronger indicator of memory accuracy than predictive JOLs; high postdictive confidence was indicative of high accuracy; and this was true for both same- and cross-race recognition memory.

**Electronic supplementary material:**

The online version of this article (10.1186/s41235-018-0125-4) contains supplementary material, which is available to authorized users.

## Significance

Eyewitness identification is often critical for solving crimes and is commonly used as evidence in legal trials. Thus, it is important to find factors that could assist the legal system in determining whether an identification is likely to be accurate or inaccurate. One such factor is an eyewitness’s expressed confidence. Shortly after viewing a crime, eyewitnesses are often asked how confident they are that they will be able to recognize the perpetrator in the future; this is *predictive confidence*. In addition, after selecting someone from a lineup, eyewitnesses are often asked how confident they are that they identified the correct person as the perpetrator; this is *postdictive confidence*. Are these confidence ratings indicative of actual accuracy? And how do they compare? In our study, we tested whether predictive and postdictive confidence ratings accurately reflect memory for same- and cross-race faces, stimuli selected because of known differences in memory strength. We found that postdictive confidence was a better indicator of memory accuracy than predictive confidence for both same- and cross-race faces. These results suggest that when possible, investigators should collect eyewitness confidence at the time of identification (under testing conditions free of contamination) as it will help investigators and jurors assess the reliability of an identification. The practice of asking eyewitnesses at the scene how likely it is that they will be able to identify the perpetrator in the future and then using these predictions as an indicator of eyewitness accuracy is discredited by our findings.

## Background

Eyewitness confidence is frequently used to determine the accuracy of eyewitness memory. However, metamemory judgments of confidence can be assessed at numerous times and how well these judgments predict eyewitness accuracy is likely to vary. In the legal system, the focus has been on eyewitness confidence collected at the time of the identification test (i.e. postdictive confidence) because what happens at this stage is typically better documented. Under unbiased testing conditions, eyewitnesses can accurately assess the strength of their memories at test, with high confidence indicative of high accuracy. These unbiased testing conditions include using a double-blind procedure, showing a fair lineup, and collecting confidence immediately after the initial identification (Wixted & Wells, 2017). In addition, semi-contaminated testing conditions also produce a reliable confidence-accuracy relationship, although the utility of a high confidence identification is still better in unbiased than biased testing conditions (Mickes, Clark, & Gronlund, 2017; Wixted & Wells, 2017).

More important, estimator variables reported to affect discrimination accuracy (i.e. the ability to distinguish between an old versus new face), such as exposure duration, retention interval, and face race, in fact do not affect the reliability of the postdictive confidence-accuracy relationship (Dodson & Dobolyi, 2015; Nguyen, Pezdek, & Wixted, 2017; Palmer, Brewer, Weber, & Nagesh, 2013; Semmler, Dunn, Mickes, & Wixted, 2018). In these studies, although overall *discrimination accuracy* was higher under better encoding conditions (e.g. longer rather than shorter exposure time, same-race rather than cross-race face), the *reliability* (i.e. the probability that an identification is likely to be correct) of a high confidence judgment did not significantly differ for faces presented under better versus poorer encoding conditions.

It is important to differentiate between these two different measures, discriminability and reliability, because each measure reflects different aspects of memory accuracy and addresses different research questions. Whereas assessing discriminability is most informative when testing the effects of system variables on eyewitness memory, assessing reliability is most informative when testing the effects of estimator variables on eyewitness memory (Mickes, 2015). Discrimination accuracy informs policymakers about best practices for collecting an eyewitness identification (i.e. methods that will increase the rate of correct identifications and decrease the rate of incorrect identifications). On the other hand, when judges and jurors are presented with eyewitness evidence in trial, they are most concerned with the issue of *reliability* (i.e. given that an eyewitness identified the suspect with a particular level of confidence, how likely is it that the identified suspect is guilty?). Thus, when deciding whether an eyewitness’s identification is trustworthy, it is a forensically important finding that estimator variables have been reported not to affect the reliability of eyewitness memory at high postdictive confidence. In other words, postdictive confidence is a useful indicator of eyewitness reliability, regardless of whether the encoding conditions were more or less optimal (Semmler et al., 2018).

Although confidence at test can be informative of the reliability of memory, in legal settings, postdictive confidence is not always well documented or uniformly collected. When confidence *is* collected at test, it is often done under biased testing conditions such as a non-blind lineup (Wixted & Wells, 2017). In fact, according to the Police Executive Research Forum (2013), although most of the surveyed agencies reported collecting eyewitness confidence during lineup identifications, they reported that a non-blind lineup procedure is used in 60% of photo lineups and in 92.1% of live lineups. Furthermore, it is unclear whether confidence is typically collected at the initial identification test rather than at a subsequent identification test. And, like other types of forensic evidence, eyewitness confidence can be contaminated by external sources making it an unreliable indicator of memory accuracy. In cases where *postdictive confidence* at test is not available, not well documented, or has been contaminated, a better indicator of eyewitness reliability might be an eyewitness’s *predictive confidence*, that is, their metamemory judgment made near the time that the perpetrator was observed. Shortly after witnessing a crime, eyewitnesses are often asked how likely it is that they will be able to identify the perpetrator in the future. Officers on the scene trying to determine which eyewitnesses are worth pursuing are likely to consider these predictions to be useful indicators of subsequent eyewitness reliability. How do the accuracy of predictive and postdictive confidence judgments compare? And do estimator variables reported to affect discrimination accuracy also affect the reliability of the predictive and postdictive confidence-accuracy relationship? This is the focus of our study.

Past research on predictions of memory performance has focused on judgments of learning (JOLs); thus, we will adopt the term JOL to refer to predictive confidence in this paper. A low JOL indicates that an item is unlikely to be remembered and a high JOL indicates that an item is highly likely to be remembered. Nelson and Dunlosky (1991) reported that JOLs tend to be more accurate when made after a brief delay than when made immediately after encoding a stimulus, a finding termed the delayed-JOL effect. In the study by Nelson and Dunlosky (1991), the delayed JOL occurred approximately 4 min after each target item, although delays of 30 s have been shown to produce a delayed-JOL effect as well (see Rhodes & Tauber, 2011 for a meta-analytic review). Nelson and Dunlosky hypothesized that whereas immediate JOLs rely more on information from short-term memory, delayed JOLs rely more on information from long-term memory and thus more closely match the context of testing. When making delayed JOLs, people are likely to engage in retrieval of the target items (as in a recall task) or the feelings of familiarity of the target items (as in a recognition task) from long-term memory, similar to what they would do during the memory test. When retrieval of the target items from long-term memory is successful and requires low effort, people would consequently provide a high JOL. In contrast, when retrieval of the target items from long-term memory is difficult or unsuccessful, people would consequently provide a low JOL.

Similar to the confidence-accuracy relationship for postdictive judgements of eyewitness memory, it is important as well to examine the confidence-accuracy relationship for predictive judgments of eyewitness memory. In a meta-analysis of nine studies, Cutler and Penrod (1989) reported that predictive confidence was only weakly correlated with subsequent identification accuracy and predictive confidence was less accurate than postdictive confidence. However, none of the studies included in the meta-analysis assessed the predictive confidence-accuracy relationship as a function of specific estimator variables, a theoretically and forensically important research question addressed in the current study. Furthermore, Cutler and Penrod examined the confidence-accuracy relationship using correlations and did not assess eyewitness reliability at specific levels of confidence. Since the meta-analysis, researchers (Juslin, Olsson, & Winman, 1996; Wixted & Wells, 2017) have reported that correlational measures are an inappropriate measure of the confidence-accuracy relationship as it relates to eyewitness memory and the legal system and, instead, proposed the use of more informative measures such as confidence-accuracy characteristic (CAC) analysis (Mickes, 2015). CAC analysis is a more informative method of assessing the confidence-accuracy relationship because it addresses the question that is most forensically relevant to judges and jurors who are evaluating whether an eyewitness’s identification is trustworthy (i.e. given that an eyewitness identified the suspect with a particular level of confidence, how likely is it that the identified suspect is guilty?). In the current study, we extend past research on predictive judgments in eyewitness memory by using CAC analysis to examine the reliability of the predictive confidence-accuracy relationship across levels of an estimator variable known to affect discrimination accuracy and how it compares to the reliability of the postdictive confidence-accuracy relationship.

The central question in our study is how do immediate and delayed predictive confidence compare to postdictive confidence in predicting the reliability of eyewitness identifications? To make this assessment, we need to manipulate a variable known to affect the strength of overall memory (i.e. discrimination accuracy) and then compare confidence ratings and memory accuracy across levels of this variable. In this study, we manipulate discriminability of face memory by using same-race and cross-race faces. It is well documented that discrimination accuracy is higher for faces of the same race than of a different race as the observer, a phenomenon known as the cross-race effect (CRE; Malpass & Kravitz, 1969). Meissner, Brigham, and Butz (2005) and others (Hills & Pake, 2013; Hugenberg, Young, Bernstein, & Sacco, 2010) have argued that the CRE is a result of qualitative differences in encoding same- and cross-race faces. In our study as well as related studies discussed below, face race is simply included as a manipulation of memory strength. However, these findings should apply as well to any estimator variables that affect discrimination accuracy, variables that are likely to influence eyewitness memory in real-world scenarios.

Hourihan, Benjamin, and Liu (2012) tested whether the CRE affects the reliability of predictive JOLs. They assessed White and Asian observers’ accuracy of immediate predictive JOLs using the measure *d*_a_ (Benjamin & Diaz, 2008). Using only an immediate measure of JOLs (JOLs were presented 1 s after offset of each stimulus), they reported that whereas White observers were more accurate predicting memory performance for White than Asian faces, Asian observers were similarly accurate predicting memory performance for White and Asian faces. And, most important, overall predictive metamemory accuracy was low (*M*_*d*a_ = 0.32 and *M*_*d*a_ = 0.34 for White and Asian observers, respectively; values close to zero indicate low metamemory accuracy), indicating that when predictive judgments take place immediately after stimulus presentation, people tend to have poor metamemory insight into their future face recognition ability for both same- and cross-race faces.

However, based on the findings of Nelson and Dunlosky (1991) with both immediate and delayed tests of JOLs, it is predicted that for White participants, although the reliability of immediate JOLs for cross-race faces is low, the reliability of delayed JOLs for cross-race faces would be higher. This is because observers rely on information from their short-term memory when making immediate JOLs, but from relatively more long-term memory when making delayed JOLs. Due to qualitative differences in encoding, the forgetting of information in memory over time is predicted to be faster for cross- than same-race faces (Deffenbacher, Bornstein, McGorty, & Penrod, 2008). If observers perceive this difference, then predictive metamemory judgments for cross-race faces will be more accurate after a brief delay than when made immediately. We test this hypothesis in our study.

Because we are interested in evaluating metamemory accuracy and its application to eyewitness identification, it is important to use measures that are most informative to the legal system, for example, CAC curves (Mickes, 2015). Assessing CAC curves is a similar approach to calibration analyses (Juslin et al., 1996) and has been utilized to assess the reliability of the *postdictive* confidence-accuracy relationship. CAC curves have not yet been used to assess the reliability of the *predictive* confidence-accuracy relationship and our study will address this issue. In examining CAC curves, the proportion correct (the number of correct identifications divided by the number of total identifications) is plotted for each level of JOL or confidence. Although observers may have better overall predictive metamemory accuracy for same- than cross-race faces (Hourihan et al., 2012), the reliability of high predictive JOLs may not differ between same- and cross-race faces (or for other estimator variables) and this has not been examined previously.

In two experiments, we test the relative utility of predictive versus postdictive metamemory judgments and whether the reliability of these metamemory judgments changes across an estimator variable known to vary in discrimination accuracy, same- versus cross-race faces. Experiment 2 is a replication of Experiment 1 using a different sample of participants and a different sample of stimulus faces. The experimental procedure and statistical analyses were identical across both experiments. Although we analyzed the data for Experiment 1 and Experiment 2 separately, for brevity, we report the results for both experiments together. In addition, to simplify and clarify the presentation of the primary results, we report the secondary results in footnotes and Additional file 1.

## Methods

### Experiments 1–2

#### Participants and design

A total of 244 White participants (Experiment 1) and 241 White participants (Experiment 2) who live in the U.S. were recruited from Amazon’s Mechanical Turk.^1^ After cleaning the data (see Additional file 1 where this procedure is clarified), 53 participants in Experiment 1 and 43 participants in Experiment 2 were excluded from the analyses, with the final *N*_*Exp.1*_ = 191; *N*_*Exp.2*_ = 198. The study was a 2 (Face Race: White or Black) × 2 (JOL type: immediate or delay) mixed factorial design with face race manipulated within-subjects. Analyses were conducted on: (1) discrimination accuracy, measured by *d*´; (2) predictive metamemory accuracy, measured by proportion correct; (3) postdictive metamemory accuracy, measured by proportion correct; and (4) the association between predictive and postdictive metamemory judgments, measured by regression coefficients.

#### Materials and procedure

The experiment was conducted on Qualtrics. Face stimuli were obtained from a database of male faces used by Meissner et al. (2005). Two sets of 40 faces were selected at random from this database. The first set of 40 faces (20 White and 20 Black) was used in Experiment 1; the second set was used in Experiment 2. Meissner et al. provided two different headshots of each person: (1) smiling and wearing a casual shirt – used as study stimuli; and (2) neutral facial expression and dressed in a similar maroon colored shirt – used as test stimuli. Across participants, each face equally often served as a target (old) and a foil (new) face.

Participants were randomly assigned to either the immediate or the delayed JOL condition. In the study phase, participants viewed 10 White and 10 Black faces presented one at a time for 3 s. The order of the faces presented was randomized for each participant. After viewing each face, participants made a JOL either immediately or after a 30-s delay. We chose a delay of 30 s because we wanted to minimize participant fatigue and, importantly, the delayed-JOL effect is reported to still occur over short delay intervals of less than 1 min, although of a smaller magnitude compared to longer JOL delay intervals of several minutes (Rhodes & Tauber, 2011).

In making each JOL response, participants were asked to “Please indicate how likely you think it is that you will later recognize the face you just studied” on a 6-point Likert scale in increments of 20 (i.e. 0, 20, 40, 60, 80, or 100), with 0 being “*I am sure that I will NOT remember this face*” and 100 being “*I am sure that I WILL remember this face*.” Immediately after viewing each face, those in the immediate JOL condition were asked to make their JOL and then complete a 30-s distractor task in which they generated as many items as possible for a variety of different semantic categories.^2^ Those in the delayed-JOL condition completed the JOL and distractor task in reverse order; that is, after viewing each face, those in the delayed-JOL condition were first asked to complete the same 30-s distractor task and then make their JOL. At the end of all 20 study trials (with a JOL and distractor task for each face trial), all participants completed 30 s of the same distractor task for before beginning the test phase. Thus, the total length of the study phase was the same in the immediate and delayed-JOL conditions.

The test phase followed immediately (on average, the time between the last studied face and the first test face was 1 min). In the test phase, participants completed an old/new recognition memory test on 40 test faces (20 White and 20 Black), half old and half new. The order of faces presented was randomized for each participant. After making an old/new judgment for each test face, participants were asked “How confident are you that your old/new judgment is accurate?” and responded on a similar 6-point scale that had been used to make JOL judgments (i.e. 0, 20, 40, 60, 80, 100) with 0 being “*not at all accurate*” and 100 being “*completely accurate*.”

## Results

### Assessing recognition memory accuracy

We assessed discrimination accuracy (*d*´) to ensure that the manipulation of face race was successful in eliciting a CRE, and it was. A correction of 0.5/n and (*n* – 0.5)/*n* was applied to hit (HR) and false alarm (FAR) rates of 0 and 1, respectively (Stanislaw & Todorov, 1999). Mean *d*´, FAR, and HR values for each experimental condition are presented in Table 1. Standard error of the mean is presented in parentheses throughout the paper (both in the tables and in text). Table 2 displays the statistics for the analyses of variance (ANOVAs) on these three measures of recognition memory accuracy. The focus of these analyses is on the *d*´ data and these are presented below. Analyses of FAR and HR data are reported in the Additional file 1.

**Table 1:** Mean (SE) d’ values, false alarm rates (FAR), and hit rates (HR) per experimental condition

JOL type									
Exp.	Face Race	Immediate				Delayed			
		*d’*	FAR	HR	*N*	*d’*	FAR	HR	*N*
1	Same-Race	0.94 (0.06)	0.23 (0.02)	0.54 (0.02)	92	1.33 (0.07)	0.16 (0.01)	0.57 (0.02)	99
	Cross-Race	0.92 (0.06)	0.24 (0.02)	0.54 (0.02)		1.03 (0.06)	0.22 (0.01)	0.56 (0.02)	
2	Same-Race	1.28 (0.07)	0.16 (0.02)	0.56 (0.02)	101	1.51 (0.07)	0.18 (0.02)	0.65 (0.02)	97
	Cross-Race	0.98 (0.07)	0.21 (0.02)	0.52 (0.02)		1.01 (0.07)	0.23 (0.02)	0.55 (0.02)	

These descriptive statistics indicate that on all three measures of recognition memory accuracy, the size of the Cross-Race Effect was greater and more consistent in Experiment 2 than Experiment 1

**Table 2:** Results from three separate 2 (Face Race) × 2 (JOL type, Immediate vs Delayed) ANOVAs on d’ values, false alarm rates (FAR), and hit rates (HR)

Exp.	Effect	*d*´			*FAR*			*HR*		
		*F*	*df*	${\eta}_p^2$	*F*	*df*	${\eta}_p^2$	*F*	*df*	${\eta}_p^2$
1	Face Race	6.52^a^	1, 189	0.03	5.77^a^	1, 189	0.03	0.26	1, 189	0.001
	JOL type	10.44^b^		0.05	5.75^a^		0.03	1.39		0.01
	Face Race × JOL type	5.08^a^		0.03	5.35^a^		0.03	0.17		0.001
2	Face Race	42.15^c^	1, 196	0.18	14.34^c^	1, 196	0.07	21.78^c^	1, 196	0.10
	JOL type	2.57		0.01	1.08		0.01	5.33^a^		0.03
	Face Race × JOL type	2.62		0.01	0.03		< 0.001	2.83		0.01

df degrees of freedom per test

Effect sizes reported are partial-eta squared. These statistics indicate that on all three measures of recognition memory accuracy, the effect size for the Cross-Race Effect was greater in Experiment 2 than Experiment 1

^a^*p* < 0.05, ^b^*p* < 0.01, ^c^*p* < 0.001

In Experiments 1 and 2, a 2 (Face Race: White or Black) × 2 (JOL type: immediate or delay) ANOVA performed on *d*´ data yielded a significant main effect of face race with higher discrimination accuracy for same-race (*M*_*Exp.1*_ *=* 1.14 [0.06]; *M*_*Exp.2*_ = 1.40 [0.05]) than cross-race faces (*M*_*Exp.1*_ *=* 0.98 [0.04]; *M*_*Exp.2*_ = 0.99 [0.05]), indicating the presence of a CRE in both experiments, with a stronger effect size in Experiment 2 than Experiment 1, as reported in Table 2. In Experiment 1, there was also a significant main effect of JOL type on *d*´ with higher discrimination accuracy for faces followed by a delayed, *M =* 1.18 (0.05), than an immediate JOL, *M =* 0.93 (0.06), and a significant interaction between face race and JOL type. For same-race faces, discrimination accuracy was significantly higher in the delayed, *M =* 1.33 (0.07), than immediate JOL condition, *M =* 0.94 (0.09), *t*(189) = − 3.54, *p* < 0.001, 95% CI of the difference [− 0.61, − 0.17,], *d* = − 0.51; however, for cross-race faces, discrimination accuracy was similar in the delayed, *M =* 1.03 (0.06), and immediate JOL conditions, *M =* 0.92 (0.06), *t*(189) = − 1.29, *p* = 0.20, 95% CI of the difference [− 0.28, 0.06,], *d* = − 0.18. The main effect of JOL type and the interaction were non-significant in Experiment 2.

Together, these results indicate that the manipulation of face race was successful in varying the strength of overall memory in both experiments. Furthermore, as reported in Table 2, compared to Experiment 1, the size of the CRE was larger in Experiment 2, allowing us to compare the reliability of the JOL-accuracy and confidence-accuracy relationship across experiments, where the differences in discrimination accuracy varied.

### Assessing the accuracy of predictive metamemory judgments (JOLs)

Analyses of the accuracy of JOLs focused on responses to *old* test faces (i.e. faces participants had previously studied), because JOLs were only made for *old* faces presented in the study phase. To assess whether we replicated the findings of Hourihan et al. (2012), we ran a mixed effects logistic regression only on participants in the immediate JOL condition.^3^ Statistics for this analysis are presented in Table 3. Examining only immediate JOLs, whereas Hourihan et al. (2012) reported more accurate JOLs for same- than cross-race faces, we found that in both Experiments 1 and 2, the JOL-accuracy relationship was not different for same- versus cross-race faces. The discrepancy between our findings and those of Hourihan et al. may be due to the fact that overall *d*´ values were exceptionally high in the Hourihan study and relatively lower in our study (although well above chance). Participants in our study may have perceived the face recognition memory task to be difficult overall and thus may have been conservative with their JOL ratings. In fact, overall, participants in our study were less likely to provide high ratings of JOLs; this was true for both same- and cross-race faces (see Additional file 1 for JOL frequency distribution).

**Table 3:** Results from a multilevel model analysis for the immediate JOL condition

Exp.	Effect	Estimate	*z*	*p*
1	Face Race	0.03 (0.29)	0.10	0.92
	JOL level	0.17 (0.06)	2.79	0.01
	Face Race × JOL level	− 0.01 (0.08)	− 0.12	0.91
2	Face Race	− 0.02 (0.31)	− 0.07	0.94
	JOL level	0.23 (0.06)	3.76	< .001
	Face Race × JOL level	0.06 (0.08)	0.72	0.47

Estimates are log odds with standard error (SE) in parentheses

**Fig. 1:**
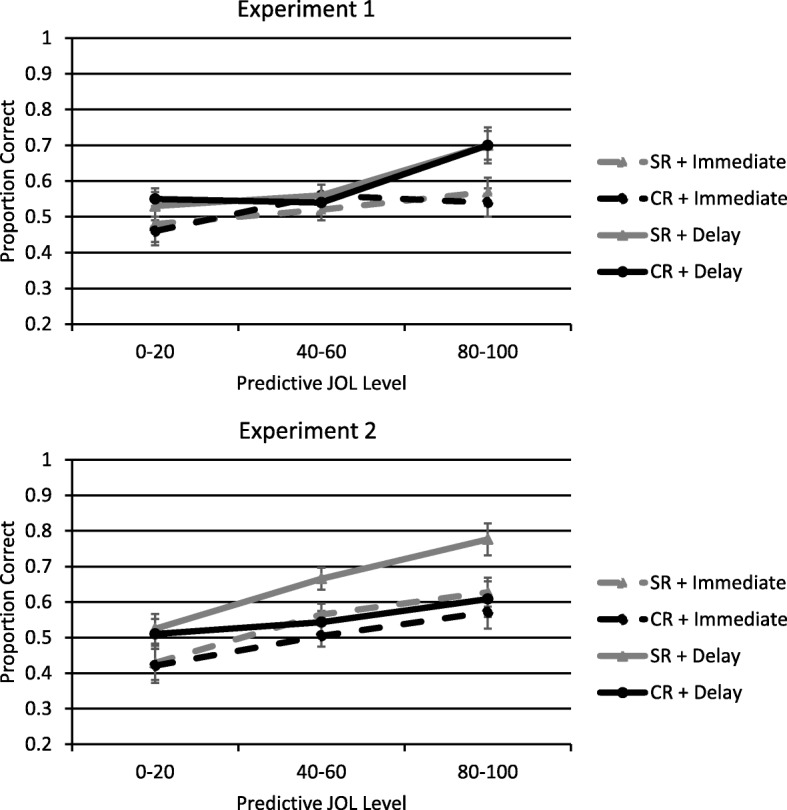
Predictive JOL-accuracy curves for Experiment 1 (*top*) and Experiment 2 (*bottom*). Proportion correct [# hits / (# hits + # misses)] is reported at each level of predictive JOL per experimental condition. *Error bars* represent standard error. SR same-race face condition, CR cross-race face condition

More important, to address our research question of whether the reliability of the predictive JOL-accuracy relationship varies as a function of face race, we compared the reliability of immediate and delayed JOLs using measures that would be most informative to the legal system (Mickes, 2015). We plotted the average proportion correct [# hits_J_ / (# hits_J_ + # misses_J_)] for each JOL level, where *J* indicates that the hits and misses were made with a specific level of JOL (see Fig. 1). These JOL-specific accuracy curves and the proportion correct reveal how reliable predictive judgments are at each JOL level. Because there were too few observations at each of the six JOL levels, for all analyses of proportion correct, we collapsed across ratings of 0 and 20 to create a “low” JOL level, across ratings of 40 and 60 to create a “medium” JOL level, and across ratings of 80 and 100 to create a “high” JOL level. Thus, the JOL scale became an aggregate of three levels (low, medium, and high). This is commonly done to create more stable estimates of proportion correct when there are too few responses made at a particular JOL or confidence level (see for example, Wixted, Mickes, Clark, Gronlund, & Roediger III, 2015). For each analysis, we removed participants who made zero ratings at each JOL level for either same-race and cross-race faces; therefore, the sample sizes across all tests were slightly different.

**Are high predictive JOLs indicative of higher accuracy than medium or low predictive JOLs, and does the reliability of the predictive JOL-accuracy relationship vary by face race or JOL type (immediate versus delay)?** To answer these questions, for each experiment, we conducted two ANOVAs on the proportion of test faces correctly recognized (using the measure specified above). Figure 1 displays the average proportion correct per experimental condition for this analysis. We did not conduct one overall analysis with all three levels of JOL (low [0–20], medium [40–60], high [80–100]), as there would have been too few participants who had non-missing data (i.e. a calculable proportion correct with non-zero values) across *all* JOL levels. For these analyses, a Bonferroni correction of α = 0.025 (0.05 / # of ANOVAs) was used.

First, the 2 (Face Race: White or Black) × 2 (JOL type: immediate or delay) × 2 (JOL Level: low [0–20] or high [80–100]) ANOVA on proportion correct in each experiment yielded a significant main effect of JOL level in both Experiment 1, *F*(1, 39) = 11.50, *p =* 0.002, ${\eta}_p^2$ = 0.23, and Experiment 2, *F*(1, 39) = 8.39, *p =* 0.006, ${\eta}_p^2$ = 0.18. The proportion correct was significantly higher at high JOLs (*M*_*Exp.1*_ = 0.64 [0.04]; *M*_*Exp.2*_ = 0.65 [0.04]) than at low JOLs (*M*_*Exp.1*_ = 0.44 [0.05]; *M*_Exp.2_ = 0.47 [0.05]). No other effects were significant in either experiment.

Next, the 2 (Face Race: White or Black) × 2 (JOL type: immediate or delay) × 2 (JOL Level: medium [40–60] or high [80–100]) ANOVA on proportion correct yielded a significant main effect of JOL level in both Experiment 1, *F*(1, 92) = 17.56, *p* < 0.001, ${\eta}_p^2$ = 0.16, and Experiment 2, *F*(1, 102) = 8.74, *p* = 0.004, ${\eta}_p^2$ = 0.08. The proportion correct was significantly higher at high JOLs (*M*_*Exp.1*_ = 0.63 [0.02]; *M*_*Exp.2*_ = 0.65 [0.03]) than at medium JOLs (*M*_*Exp.1*_ = 0.51 [0.02]; *M*_*Exp.2*_ = 0.56 [0.02]).

There were some discrepancies between the two experiments in the analyses of high versus medium JOLs. In Experiment 1, there was a significant interaction between JOL Level and JOL type, *F*(1, 92) = 5.45, *p* = 0.02, ${\eta}_p^2$= 0.06; when participants made immediate JOLs, the proportion correct was similar for high JOLs, *M* = 0.57 (0.03), and medium JOLs, *M* = 0.51 (0.03), *t*(48) = 1.36, *p* = 0.18, *d* = 0.19. However, when participants made delayed JOLs, as predicted, the proportion correct was significantly higher for high JOLs, *M* = 0.69 (0.04), than medium, JOLs, *M* = 0.51 (0.03), *t*(44) = 4.47, *p* < 0.001, *d* = 0.67. These results support the delayed-JOL effect; JOLs were more accurate if made after a brief delay than immediately. In Experiment 2, this interaction was non-significant, but there was a significant main effect of face race, *F*(1, 102) =11.01, *p* = 0.001, ${\eta}_p^2$ = 0.10. The proportion correct was significantly higher for same-race, *M* = 0.65 (0.02), than cross-race faces, *M* = 0.56 (0.02). There may not have been a face race effect on proportion correct in Experiment 1 because the size of the CRE (measured by *d*´) was smaller in Experiment 1 than Experiment 2.

Across these analyses, although accuracy was higher for high JOLs than for lower JOLs, accuracy was objectively low (relative to chance performance at 0.50) even at the highest level. This suggests that predictive metacognitive assessments are likely to be of limited utility for making real-world predictions of accuracy. Follow-up mixed effects logistic regression analyses replicated the results from the above two ANOVAs (see Additional file 1).

**Is a high predictive JOL indicative of high accuracy for both same-race and cross-race faces, and does it depend on whether JOLs are made immediately or after a delay?** To answer these questions, for each experiment, three 2 (Face Race: White or Black) × 2 (JOL type: immediate or delay) ANOVAs were conducted on the proportion recognition correct data at each of the three levels of JOL (low, medium, and high). Figure 1 displays the average proportion correct per experimental condition for this analysis. For these analyses, a Bonferroni correction of α = 0.017 (0.05 / # of ANOVAs) was used.

The most important result here for real-world cases of eyewitness identification concern high predictive JOL responses because eyewitnesses who are more confident in their ability to make a later identification are more likely to be asked to undergo a line-up identification (Cutler & Penrod, 1989). The ANOVA on the proportion correct recognition for faces with high JOL responses yielded a significant main effect of JOL type in Experiment 1, *F*(1, 101) = 10.71, *p* = 0*.*001, ${\eta}_p^2$ = 0.10, but not in Experiment 2, *F*(1, 108) = 3.93, *p* = 0*.*05, ${\eta}_p^2$ = 0.04; the direction of the effect was consistent in both experiments. As predicted, the proportion correct was higher for faces followed by a delayed JOL (*M*_*Exp.1*_ = 0.70 [0.03]; *M*_*Exp.2*_ = 0.69 [0.04]) than an immediate JOL (*M*_*Exp.1*_ = 0.56 [0.03]; *M*_*Exp.2*_ = 0.60 [0.03]). The main effect of face race was non-significant in Experiment 1, *F*(1, 101) = 0.18, *p =* 0.67, ${\eta}_p^2$ = 0.002, but significant in Experiment 2, *F*(1, 108) = 6.39, *p =* 0.01, ${\eta}_p^2$ = 0.06. In both experiments, the proportion correct was higher for same-race (*M*_*Exp.1*_ = 0.64 [0.03]; *M*_*Exp.2*_ = 0.70 [0.03]) than cross-race faces (*M*_*Exp.1*_ = 0.62 [0.03]; *M*_*Exp.2*_ = 0.59 [0.03]). Again, the differences between results of Experiments 1 and 2 can be explained by the larger CRE in Experiment 2.^4^

These results indicate that the proportion correct recognition at high JOLs were more accurate for same- than cross-race faces in Experiment 2 but not in Experiment 1, where the size of the CRE was smaller. These results make sense given the higher *d*´ for same-race faces in Experiment 2; the sample of same-race faces used in Experiment 2 was apparently easier to remember relative to the cross-race faces, thus resulting in a same-race advantage in predictions of memory performance. Furthermore, the accuracy of immediate JOLs made at even the highest level was low (*M*_*Exp.1*_ = 0.56; *M*_*Exp.2*_ = 0.60). Specifically, participants who made immediate JOLs tended to be overconfident in their future ability to remember target faces, consistent with the interpretation that they were more likely to rely on information from short-term than long-term memory. Although the interaction between face race and JOL type was not significant in Experiment 2, where the size of the CRE was larger, a comparison of the effect sizes indicates that delaying the JOL improved accuracy for same-race faces (*M*_*delayed*_ = 0.78 [0.05], *M*_*immediate*_ = 0.63 [0.04], *d* = 0.47) more than cross-race faces (*M*_*delayed*_ = 0.61 [0.05], *M*_*immediate*_ = 0.57 [0.05], *d* = 0.11).

We also performed analyses on frequency data (i.e. the number of times participants provided high versus low JOL ratings to same- and cross-race faces) and found that in Experiment 1, participants provided low JOL ratings more frequently to cross- than same-race faces, and in Experiment 2, participants provided high JOL ratings more frequently to same- than cross-race faces. These results support the notion that participants were metacognitively aware that cross-race faces were going to be more difficult to recognize in the future than same-race faces (see Additional file 1).

### Assessing the accuracy of postdictive metamemory confidence judgments

**Fig. 2:**
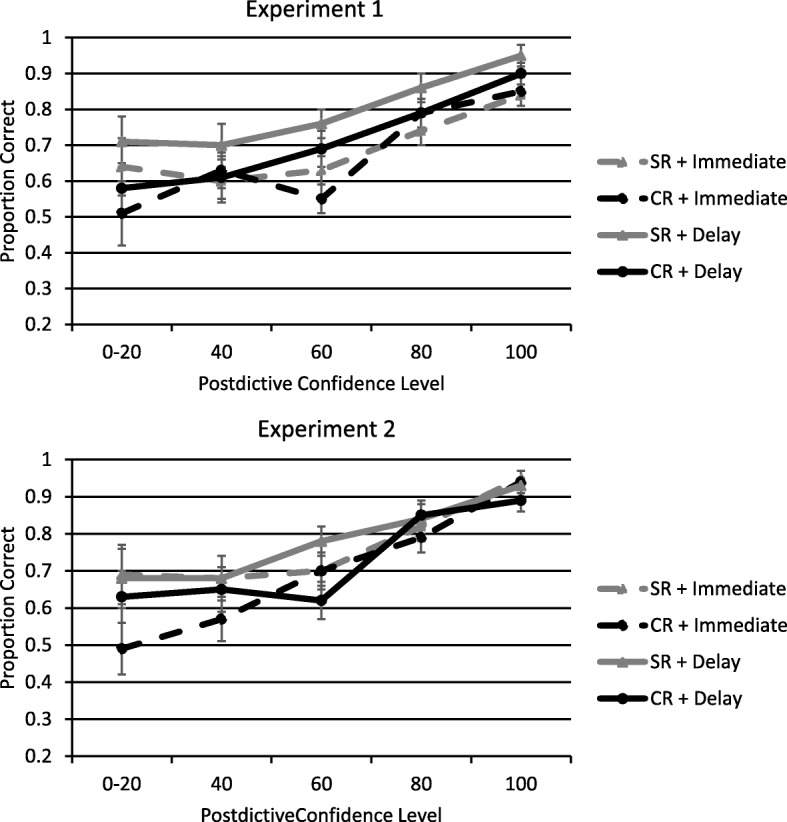
Postdictive confidence-accuracy curves for Experiment 1 (*top*) and Experiment 2 (*bottom*). Proportion correct [# hits / (# hits + # false alarms)] is reported at each level of postdictive confidence per experimental condition. *Error bars* represent standard error. SR same-race face condition, CR cross-race face condition

We next analyzed the results for ratings of postdictive confidence to assess the reliability of the postdictive confidence-accuracy relationship. For this set of analyses, we focused on “old” responses (i.e. faces that participants said they had previously studied), similar to analyzing the data only for “choosers” in an eyewitness identification paradigm. Consistent with previous research on the CA relationship, we plotted the average proportion correct [# hits_c_/(# hits_c_ + # false alarms_c_)] for each level of confidence, where *C* indicates that the hits and false alarms were made with a specific level of confidence. Thus, the proportion correct at each level of postdictive confidence is the probability that a positive identification of “old” is accurate. These results are displayed in Fig. 2. Because there were too few observations at the two lowest levels of confidence, for all analyses of proportion correct for postdictive confidence we collapsed across confidence ratings of 0 and 20; thus, the final confidence scale consisted of five levels (0–20, 40, 60, 80, and 100). For each analysis, we removed participants who made zero ratings at each confidence level for same-race faces and for cross-race faces; therefore, the sample sizes were slightly different in all tests (total *N*_*Exp.1*_ = 191; *N*_*Exp.2*_ = 198).

**Is high postdictive confidence indicative of higher accuracy than the lower levels of postdictive confidence, and does the reliability of the confidence-accuracy relationship vary by face race or immediate versus delayed JOL type?** To answer these questions, for each experiment, we conducted four ANOVAs to compare the proportion correct at the highest confidence level (100) to the proportion correct at each of the lower four confidence levels (0–20, 40, 60, 80). Figure 2 displays the average proportion correct per experimental condition for this analysis. We did not conduct one overall 2 (Face Race: White or Black) × 2 (JOL type: immediate or delay) × 5 (confidence level: 0–20, 40, 60, 80, 100) ANOVA on the proportion correct data as there would have been too few participants who had non-missing data (i.e. a calculable proportion correct with non-zero values) across *all* confidence levels. For these four analyses, a Bonferroni correction of α = 0.0125 (0.05 / # of ANOVAs) was used.

First, we found that higher postdictive confidence was indicative of higher recognition memory accuracy than lower postdictive confidence. A 2 (Face Race: White or Black) × 2 (JOL type: immediate or delay) × 2 (confidence level: 0–20 or 100) ANOVA of the proportion correct yielded a significant main effect of confidence level in both Experiment 1, *F*(1, 24) = 15.58, *p =* 0.001, ${\eta}_p^2$ = 0.39, and Experiment 2, *F*(1, 21) = 45.92, *p* < 0.001, ${\eta}_p^2$ = 0.69. No other effects were significant. These results indicate that the proportion correct at the highest level of confidence (*M*_*Exp.1*_ = 0.85 [0.05]; *M*_*Exp.2*_ = 0.93 [0.04]) was significantly higher than at the lowest confidence level (*M*_*Exp.1*_ = 0.58 [0.06]; *M*_*Exp.2*_ = 0.51 [0.06]); this did not vary with race or JOL type. Subsequent ANOVAs comparing each of the other three lower confidence levels to confidence ratings of 100 yielded similar results.^5^

Across these analyses, it is evident that compared to face race and JOL type, postdictive confidence is the stronger predictor of accuracy, with postdictive confidence accounting for a larger amount of the variability in proportion correct. Most important, high confidence (100), when compared to all lower levels of confidence, was indicative of higher accuracy; this did not vary with race or JOL type. Follow-up mixed effects logistic regression analyses replicated results from the ANOVAs (see Additional file 1). Together, these findings replicate our previous work on the confidence-accuracy relationship in same- and cross-race faces (Nguyen et al., 2017).

**Is high postdictive confidence indicative of high accuracy for both same-race and cross-race faces, and does it depend on whether participants had made immediate or delayed JOLs before test?** It is important to include JOL type as a factor in the analysis of postdictive confidence because in Experiment 1, there were differences in *d*´ between the immediate and delayed JOL conditions. For each experiment, five 2 (Face Race: White or Black) × 2 (JOL type: immediate or delay) ANOVAs were conducted on the proportion correct data at each of the five levels of confidence. Figure 2 displays the average proportion correct per experimental condition for this analysis. For these analyses, a Bonferroni correction of α = 0.01 (0.05 / # of ANOVAs) was used.

Analyses of the proportion correct for confidence ratings of 100 yielded non-significant main effects of face race [*F*_*Exp.1*_(1, 111) = 0.61, *p =* 0.44, ${\eta}_p^2$ = 0.01; *F*_*Exp.2*_(1, 113) = 1.43, *p* = 0.23, ${\eta}_p^2$ = 0.01], JOL type [*F*_*Exp.1*_(1, 111) = 4.58, *p* = 0.04, ${\eta}_p^2$ = 0.04; *F*_*Exp.*2_(1, 113) = 2.07, *p* = 0.15, ${\eta}_p^2$ = 0.02], and their interaction [*F*_*Exp.1*_(1, 111) = 0.98, *p* = 0.32, ${\eta}_p^2$ = 0.01; *F*_*Exp.2*_(1, 113) = 0.55, *p* = 0.46, ${\eta}_p^2$ = 0.01]. The three ANOVAs on the proportion correct for confidence ratings of 80, 40, and 0–20 for Experiment 1 and ratings of 80, 60, 40, and 0–20 for Experiment 2 also yielded no significant effects. However, in Experiment 1, analyses of the proportion correct for confidence ratings of 60 yielded a significant main effect of JOL type, *F*(1, 123) = 10.24, *p* = 0.002, ${\eta}_p^2$ = 0.08, with a higher proportion correct for faces followed by a delayed, *M* = 0.73 (0.03), than an immediate JOL, *M* = 0.59 (0.03). No other effects were significant.

In the above analyses, the most important result for real-world cases of eyewitness identification concern high postdictive confidence responses of 100, because eyewitnesses who make identifications at the highest level of confidence are most likely to testify at trial and to do so confidently. We found that at a high level of postdictive confidence, same-race and cross-race recognition judgments were similarly accurate (Cohen’s *d*_*Exp.1*_ = 0.07; *d*_*Exp.2*_ = 0.11), suggesting that participants are metacognitively able to accurately adjust their postdictive judgments to account for any encoding differences between same- and cross-race faces. Although the CRE (as measured by *d*´) was larger in Experiment 2 than Experiment 1 (*d*_*Exp1*_ = 0.16; *d*_*Exp2*_ = 0.42), the CRE at high postdictive confidence in both Experiments 1 and 2 were of a similar magnitude. Importantly, as evidenced by the small Cohen’s *d* values reported above, the size of the CRE at high postdictive confidence in both experiments was minimal. This finding replicates our previous work on the postdictive confidence-accuracy relationship in memory for same- and cross-race faces (Nguyen et al., 2017). Furthermore, in contrast to predictive JOLs, the proportion correct recognition for postdictive confidence was objectively high (relative to chance performance at 0.50) (see Fig. 2). This suggests that postdictive judgments are more accurate than predictive judgments, which is supported by later analyses.

Similar to the analysis of frequency data for predictive JOLs, we also compared the frequency of postdictive confidence ratings for same- versus cross-race faces (see Additional file 1). These results suggest that although participants more frequently provided high confidence ratings to same- than cross-race faces, when participants *did* provide high confidence ratings for cross-race faces, they were as accurate as when they provided high confidence ratings for same-race faces. This also indicates that participants had some metacognitive awareness of a memory deficit for cross-race faces because they were aware that they should not be highly confident as often for cross-race than same-race faces and were more likely to rate low postdictive confidence for cross-race than same-race faces.

### The relative utility of predictive JOLs versus postdictive confidence in predicting memory accuracy

To address the critical research question, how does the reliability of predictive and postdictive confidence judgments compare, we ran a mixed effects logistic regression model for each experiment. For this analysis, we focused on responses to *old faces* (i.e. target faces participants had previously studied). Although analyses of confidence typically focus on *“old” responses* (which may include both old and new test *faces*), because JOLs are made only for *old faces* presented in the study phase, we wanted to compare the predictive power of JOLs and postdictive confidence on the same subset of faces, with accuracy coded as 0 = miss and 1 = hit.

The model consisted of random intercepts of participants and face race, JOL type, JOL level, and confidence as predictors. JOL level and confidence were assessed at six levels: 0, 20, 40, 60, 80, and 100. Results indicated that while a statistically significant predictor of accuracy, JOLs were far less predictive when postdictive confidence was also in the model (Experiment 1: estimate_JOL_ [log odds] = 0.07 (0.03, z = 2.01, *p* = 0.04; estimate_confidence_ [log odds] = 0.47 (0.03, z = 15.66, *p* < 0.001; Experiment 2: estimate_JOL_ [log odds] = 0.17 (0.03, z = 4.98, *p* < 0.001; estimate_confidence_ [log odds] = 0.39 (0.03, z = 12.72, *p* < 0.001). Postdictive confidence was a far more reliable indicator of memory accuracy than JOLs. This is a key finding in this study.

Neither face race nor JOL type (immediate versus delayed) was a significant predictor of accuracy in Experiment 1. However, in Experiment 2, face race (estimate [log odds] = 0.24 [0.07, z = 3.52, *p* < 0.001], and JOL type (estimate [log odds] = 0.41 [0.12, z = 3.44, *p* < 0.001) were significant predictors of accuracy. This discrepancy can be explained by the fact that in Experiment 1, face race and JOL type affected discrimination accuracy of *new* faces more than *old* faces; earlier results indicated significant effects on FAR but not HR. However, in Experiment 2, there were significant effects of face race and JOL type on HR as well, in other words, on discrimination accuracy of *old* faces.

We conducted additional analyses to assess whether there was a reliable relationship between predictive JOLs and postdictive confidence.^6^ Overall, the relationship between predictive JOLs and postdictive confidence was weak and did not differ between same- and cross-race faces nor between immediate and delayed JOLs. This suggests that predictive and postdictive metamemory judgments are likely to involve different underlying cognitive processes.

## Discussion

This study examines two main issues. First, how does the reliability of immediate and delayed predictive JOLs compare, and does the reliability of these metamemory judgments differ across an estimator variable known to affect the discrimination accuracy of eyewitness memory, face race? Second, and more important, how do predictive JOLs and postdictive confidence compare in predicting the accuracy of face memory?

First, we found that high JOLs were associated with a higher proportion correct than low JOLs in the delayed but not the immediate JOL condition. However, although the proportion correct was higher for high than low levels of JOL, accuracy at the highest JOL level was still objectively low (*M*_*Exp.1*_ = 0.63; *M*_*Exp.2*_ = 0.65) (chance recognition accuracy was 0.50). Furthermore, when there is a larger CRE on discrimination accuracy (in Experiment 2), the reliability of a high predictive JOL was greater for same- than cross-race faces; delaying the JOL improved the accuracy of predictions for same-race memory more than cross-race memory. These results suggest that although delaying JOLs allow participants to evaluate their future memory performance more accurately for same- than cross-race faces, nonetheless, the utility of predictive metamemory judgments for estimating subsequent memory accuracy is limited for both same-race and cross-race faces.

We also found that in Experiment 1, whether participants had made immediate or delayed JOLs in the study phase affected their subsequent discrimination accuracy for same-race but not cross-race faces. When assessing memory for same-race faces, discrimination accuracy was significantly higher for delayed than immediate JOLs; however, when assessing memory for cross-race faces, discrimination accuracy was similar for delayed and immediate JOLs. One interpretation of this finding is that due to superior qualitative encoding of same- than cross-race faces, when making delayed JOLs, observers are more likely to engage in successful retrieval practice of same- than cross-race faces. Although there was not a significant interaction between face race and JOL type in Experiment 2, the pattern of means replicated that of Experiment 1.

Regarding our second main issue, we found that postdictive confidence reliably predicted subsequent memory accuracy; faces rated with high postdictive confidence were more likely to be accurately recognized than faces rated with lower postdictive confidence, and unlike the low proportion correct at high JOLs, the proportion correct at high postdictive confidence was objectively high (*M*_*Exp.1*_ = 0.89; *M*_*Exp.2*_ = 0.93). Importantly, as evidenced by the regression analyses, postdictive confidence was a far stronger indicator of memory accuracy than both immediate and delayed predictive JOLs. And unlike the predictive JOL-accuracy relationship, the reliability of the postdictive confidence-accuracy relationship did not differ between same- and cross-race faces. This suggests that observers are metacognitively aware of differences in encoding difficulty between same- and cross-race faces at test. This may be because when making postdictive metamemory judgments at test, observers receive more specific information about their performance (i.e. they are aware that memory weakens faster for cross- than same-race faces) and can adjust their confidence ratings more accurately. Using more informative measures of the confidence-accuracy relationship, CAC curves, this finding replicates earlier work in the domains of education (Pierce & Smith, 2001) and eyewitness memory (Cutler & Penrod, 1989) that reported higher correlations between postdictive confidence and accuracy than predictive confidence and accuracy.

Another important finding from a cognitive point of view is that overall, the relationship between predictive JOLs and postdictive confidence was weak. This finding suggests that these two types of metamemory judgments involve different underlying cognitive processes. One possible explanation for the differences in the utility of predictive and postdictive confidence is that people may be relying on different information from memory when making these two metamemory judgments. For example, although people are likely to engage in active retrieval of information from memory when making predictive JOLs, based on response time data, Tauber, Dunlosky, and Rawson (2015) postulated that predictive JOLs may not always involve a complete memory search. If people do not conduct a thorough memory search when making JOLs, then their judgments of future memory performance will be less accurate. However, people are likely to spend more time performing a thorough memory search when tested on their memory, and thus metamemory judgments provided at this time are more likely to be accurate. Although Tauber et al. (2015) did not directly compare predictive and postdictive judgments of confidence, their proposed explanation may account for why the distribution of predictive JOLs in our study was clustered around low to medium JOLs; if participants were performing an incomplete memory search when making a JOL, then they are less likely to retrieve sufficient information from memory and, consequently, provide lower predictions of future memory performance.

A second possible explanation for the low accuracy of even high predictive delayed JOLs is that although delayed JOLs rely more on information from long-term than short-term memory, the information available in memory during a delayed JOL still may not be similar to the information that is available at test, which occurs after an even longer delay from encoding. These potential explanations for the reported differences in the reliability of predictive and postdictive metamemory judgments are not exhaustive and our study does not offer a critical test of the mechanisms involved. Articulating the cognitive processing differences between predictive and postdictive metamemory judgments is a fruitful direction for future research.

## Conclusion

In summary, we found that both immediate and delayed predictive JOLs were a weak indicator of subsequent memory accuracy. In contrast, postdictive confidence was a strong indicator of subsequent memory accuracy, with high confidence associated with high accuracy. The finding that postdictive confidence is a more reliable indicator of memory accuracy than predictive JOL has applied relevance and importance to the field of eyewitness memory given that eyewitnesses are often asked to make these metamemory judgments at different times throughout their interactions with the legal system. Our findings suggest that when possible, investigators should collect judgments of eyewitness confidence at the time of the initial identification (with minimized contamination of testing procedures) as these judgments will help triers of fact assess the reliability of an identification. However, if information about eyewitness confidence at test is not available or if it has been highly contaminated, predictive judgments, collected after a brief delay, provide information somewhat prognostic of subsequent identification accuracy, information that is less useful but better than chance.

## Additional file

Additional file 1: Includes detailed information about data cleaning procedures and supplementary analyses, tables, and figures. (DOCX 57 kb)

## Abbreviations

ANOVA: Analysis of variance
CAC: Confidence-accuracy characteristic
CRE: Cross-race effect
JOL: Judgment of learning

## Footnotes

1 We did not conduct a fully-crossed race design and collected data from only White participants because the magnitude of the CRE is largest in White participants (Meissner & Brigham, 2001); therefore, if any differences exist in metacognitive ability, it should be most evident in White participants. In addition, Black and White Mechanical Turk participants are not comparable; whereas White participants make up a larger percentage of the Mechanical Turk population, Black participants are underrepresented in the Mechanical Turk population compared to the U.S. population (Paolacci & Chandler, 2014).

2 Participants had to generate as many items as they could in 30 s for the following categories: States, marine animals, fruits/vegetables, male names, book titles, furniture, kitchen items, “S” words, ice cream flavors, movie titles, celebrities, cities, song titles, office items, TV shows, sports, female males, countries, land animals, professions, “P” words.

3 The model consisted of random intercepts of participants (as judgment trials were nested within participants), and face race, JOL level, and their interaction as predictors, with accuracy coded as 0 = miss and 1 = hit. For this analysis, JOL level was assessed at six levels: 0, 20, 40, 60, 80, and 100. Results from the logistic regression assessing the accuracy of immediate JOLs indicated that JOL level was a significant predictor of recognition accuracy in both experiments; faces rated with higher JOLs were more accurately recognized. No other effects were significant. Examining only immediate JOLs, whereas Hourihan et al. (2012) reported more accurate JOLs for same- than cross-race faces, we found that the JOL-accuracy relationship was not different for same- versus cross-race faces.

4 The ANOVA on the proportion correct recognition for faces with medium JOL responses yielded no significant effects in Experiment 1, but a significant main effect of face race in Experiment 2, *F*(1, 184) = 10.79, *p* = 0.001, ${\eta}_p^2$ = 0.06. The proportion correct was significantly higher for same-race, *M* = 0.62 (0.02), than cross-race faces, *M* = 0.52 (0.02). There was also a significant main effect of JOL type in Experiment 2, *F*(1, 184) = 4.49, *p* = 0.035, ${\eta}_p^2$ = 0.02. The proportion correct was significantly higher for delayed, *M* = 0.61 (0.02), than immediate JOLs, *M* = 0.54 (0.02). In both Experiments 1 and 2, the ANOVA on the proportion correct recognition for faces with low JOL responses yielded no significant effects.

5 The 2 (Face Race: White or Black) × 2 (JOL type: immediate or delay) × 2 (confidence: 40 or 100) ANOVA of the proportion correct also yielded a significant main effect of confidence level in both Experiment 1, *F*(1, 43) = 15.47, *p* < 0.001, ${\eta}_p^2$ = 0.27, and Experiment 2, *F*(1, 42) = 82.85, *p* < 0.001, ${\eta}_p^2$ = 0.66. No other effects were significant. These results indicate that the proportion correct at the highest level of confidence (*M*_*Exp.1*_ = 0.85 [0.05]; *M*_*Exp.2*_ = 0.96 [0.02]) was significantly higher than at a confidence judgment of 40 (*M*_*Exp.1*_ = 0.66 [0.04]; *M*_*Exp.2*_ = 0.55 [0.04]); this did not vary with race or JOL type. Next, the 2 (Face Race: White or Black) × 2 (JOL type: immediate or delay) × 2 (confidence: 60 or 100) ANOVA on the proportion correct also yielded a significant main effect of confidence level in both Experiment 1, *F*(1, 66) = 40.04, *p* < 0.001, ${\eta}_p^2$ = 0.38, and Experiment 2, *F*(1, 65) = 50.92, *p* < 0.001, ${\eta}_p^2$ = 0.44. No other effects were significant. These results indicate that the proportion correct at the highest level of confidence (*M*_*Exp.1*_ = 0.89 [0.02]; *M*_*Exp.2*_ = 0.93 [0.02]) was significantly higher than at a confidence judgment of 60 (*M*_*Exp.1*_ = 0.64 [0.03]; *M*_*Exp.2*_ = 0.68 [0.03]); this did not vary with race or JOL type. Finally, a 2 (Face Race: White or Black) × 2 (JOL type: immediate or delay) × 2 (confidence: 80 or 100) ANOVA on the proportion correct also yielded a significant main effect of confidence level in both Experiment 1, *F*(1, 71) = 18.49, *p* < 0.001, ${\eta}_p^2$ = 0.21, and Experiment 2, *F*(1, 71) = 17.35, *p* < 0.001, ${\eta}_p^2$ = 0.20. No other effects were significant in this analysis. These results indicate that the proportion correct at the highest level of confidence (*M*_*Exp.1*_ = 0.89 [0.02]; *M*_*Exp.2*_ = 0.91 [0.02]) was significantly higher than at a confidence judgment of 80 (*M*_*Exp.1*_ = 0.74 [0.03]; *M*_*Exp.2*_ = 0.80 [0.03]); this did not vary with race or JOL type.

6 Is there an association between predictive JOLs and postdictive confidence judgments? If an eyewitness provides a high postdictive confidence judgment at test, is the identified suspect someone the eyewitness had previously said they were highly likely to remember (i.e. a high JOL)? In the examination of the relationship between JOLs and postdictive confidence, we focused our analysis on *hits* (i.e. *old* target faces correctly identified as “old”). This is because we want to assess the relationship between predictive and postdictive judgments on the same subset of faces. JOLs are made on *old* faces (faces previously studied, resulting in test trials that are either hits or misses) whereas postdictive confidence ratings can be made on *old* and *new* faces, with typical analyses of the confidence-accuracy relationship focusing on “old” *responses* (test faces participants had responded to as having been seen before). This is similar to analyzing data from “choosers” in an eyewitness identification paradigm and is thus forensically relevant. Thus, we computed a Spearman’s rank correlation between JOL and postdictive confidence on *hits* for each participant and analyzed the average correlation across participants in each experimental condition. Some participants were excluded from this set of analyses because their correlation could not be computed due to no variability (a standard deviation of zero) in either their JOLs or postdictive confidence judgments for *hits*. The sample sizes in all tests was thus slightly different. Table 3 in Supplementary Materials presents the mean correlation and sample size per condition. The correlation between predictive JOLs and postdictive confidence judgments was low, and similarly low for both immediate and delayed predictive JOLs. In Experiments 1 and 2, the mean correlation collapsed across all conditions was significantly different from zero (rejecting the null hypothesis of no relationship) but small, Experiment 1: *r*_s_ = 0.10, *t*(187) = 3.89, *p* < 0.001, 95% CI of the difference [0.05, 0.15]; Experiment 2: *r*_s_ = 0.20, *t*(191) = 8.47, *p* < 0.001, 95% CI of the difference [0.16, 0.25]. Furthermore, in Experiments 1 and 2, a 2 (Face Race: White or Black) × 2 (JOL type: immediate or delay) ANOVA on *r*_s_ yielded no significant differences across experimental conditions, with non-significant main effects of Face Race [*F*_*Exp.1*_(1, 158) = 1.55, *p =* 0.22, ${\eta}_p^2$ = 0.01; *F*_*Exp.2*_(1, 140) = 1.86, *p =* 0.17, ${\eta}_p^2$ = 0.01], JOL type [*F*_*Exp.1*_(1, 158) < 0.001, *p* = 0.99, ${\eta}_p^2$ < 0.001; *F*_*Exp.2*_(1, 140) = 1.71, *p* = 0.19, ${\eta}_p^2$ = 0.01], and their interaction [*F*_*Exp.1*_(1, 158) = 0.06, *p* = 0.81, ${\eta}_p^2$ < 0.001; *F*_*Exp.2*_(1, 140) = 0.10, *p* = 0.75, ${\eta}_p^2$ = 0.001].

## References

[CR1] Benjamin AS, Diaz M, Dunlosky J, Bjork RA (2008). Measurement of relative metamnemonic accuracy. Handbook of memory and metamemory.

[CR2] Cutler BL, Penrod SD (1989). Forensically relevant moderators of the relation between eyewitness identification accuracy and confidence. Journal of Applied Psychology.

[CR3] Deffenbacher KA, Bornstein BH, McGorty EK, Penrod SD (2008). Forgetting the once-seen face: Estimating the strength of an eyewitness’s memory representation. Journal of Experimental Psychology: Applied.

[CR4] Dodson CS, Dobolyi DG (2015). Confidence and eyewitness identifications: The cross-race effect, decision time and accuracy. Applied Cognitive Psychology.

[CR5] Hills PJ, Pake JM (2013). Eye-tracking the own-race bias in face recognition: Revealing the perceptual and socio-cognitive mechanisms. Cognition.

[CR6] Hourihan KL, Benjamin AS, Liu X (2012). A cross-race effect in metamemory: Predictions of face recognition are more accurate for members of our own race. Journal of Applied Research in Memory and Cognition.

[CR7] Hugenberg K, Young SG, Bernstein MJ, Sacco DF (2010). The categorization-individuation model: An integrative account of the other-race recognition deficit. Psychological Review.

[CR8] Juslin P, Olsson N, Winman A (1996). Calibration and diagnosticity of confidence in eyewitness identification: Comments on what can be inferred from the low confidence-accuracy correlation. Journal of Experimental Psychology: Learning, Memory, and Cognition.

[CR9] Malpass RS, Kravitz J (1969). Recognition for faces of own and other race. Journal of Personality and Social Psychology.

[CR10] Meissner CA, Brigham JC (2001). Thirty years of investigating the own-race bias in memory for faces: A meta-analytic review. Psychology, Public Policy, and Law.

[CR11] Meissner CA, Brigham JC, Butz DA (2005). Memory for own- and other-race faces: A dual-process approach. Applied Cognitive Psychology.

[CR12] Mickes L (2015). Receiver operating characteristic analysis and confidence-accuracy characteristic analysis in investigations of system variables and estimator variables that affect eyewitness memory. Journal of Applied Research in Memory and Cognition.

[CR13] Mickes L, Clark SE, Gronlund SD (2017). Distilling the confidence-accuracy message: A comment on Wixted and wells (2017). Psychological Science in the Public Interest.

[CR14] Nelson TO, Dunlosky J (1991). When people’s judgments of learning (JOLs) are extremely accurate at predicting subsequent recall: The “delayed-JOL effect”. Psychological Science.

[CR15] Nguyen TB, Pezdek K, Wixted JT (2017). Evidence for a confidence-accuracy relationship in memory for same- and cross-race faces. Quarterly Journal of Experimental Psychology.

[CR16] Palmer MA, Brewer N, Weber N, Nagesh A (2013). The confidence-accuracy relationship for eyewitness identification decisions: Effects of exposure duration, retention interval, and divided attention. Journal of Experimental Psychology: Applied.

[CR17] Paolacci G, Chandler J (2014). Inside the turk: Understanding mechanical turk as a participant pool. Current Directions in Psychological Science.

[CR18] Pierce BH, Smith SM (2001). The postdiction superiority effect in metacomprehension of text. Memory & Cognition.

[CR19] Police Executive Research Forum (2013). A national survey of eyewitness identification procedures in law enforcement agencies.

[CR20] Rhodes MG, Tauber SK (2011). The influence of delaying judgments of learning on metacognitive accuracy: A meta-analytic review. Psychological Bulletin.

[CR21] Semmler C, Dunn J, Mickes L, Wixted JT (2018). The role of estimator variables in eyewitness identification.

[CR22] Stanislaw H, Todorov N (1999). Calculation of signal detection theory measures. Behavior Research Methods, Instruments, & Computers.

[CR23] Tauber SK, Dunlosky J, Rawson KA (2015). The influence of retrieval practice versus delayed judgments of learning on memory. Experimental Psychology.

[CR24] Wixted JT, Mickes L, Clark SE, Gronlund SD, Roediger HL (2015). Initial eyewitness confidence reliably predicts eyewitness identification accuracy. American Psychologist.

[CR25] Wixted JT, Wells GL (2017). The relationship between eyewitness confidence and identification accuracy: A new synthesis. Psychological Science in the Public Interest.

