# Supplementary material for: Postdictive confidence (but not predictive confidence) predicts eyewitness memory accuracy
Source: Cogn Res Princ Implic. 2018 Aug 29;3:32. doi: 10.1186/s41235-018-0125-4 (PMC6113198; doi:10.1186/s41235-018-0125-4)
Supplement: Supplementary file 1 — Includes detailed information about data cleaning procedures and supplementary analyses, tables, and figures. (DOCX 57 kb) [file 41235_2018_125_MOESM1_ESM.docx]

**Data Cleaning Procedures**

An approximately equal number of participants were removed from each experimental condition (Experiment 1: 33 in the immediate JOL condition, 20 in the delayed JOL condition; Experiment 2: 17 in the immediate JOL condition, 26 in the delayed JOL condition). We excluded the following number of participants from analyses for the following reasons:

- Four participants responded with “almost none of my” to the question “I gave this experiment ___ of my attention.”
- Five participants who responded “No” to the question “Should we include your data in our analyses?”
- Twenty-eight participants who provided fewer than 2 responses on at least two of the naming filler tasks
- Ten participants who were considered univariate outliers on either *d*´ for White or Black faces (determined univariate outlier if had a standardized score more extreme than +/- 2.5)
- Two participants who were considered multivariate outliers on *d*´ (determined a multivariate outlier if Mahalanobis distance value had an associated *p*-value < .01)
- Forty-five participants who consistently responded with the same value for either all the JOLs, confidence ratings, or Old/New judgments
- Two participants who said they had technical difficulties with Qualtrics

**Additional Analyses**

**Discrimination Accuracy**

**Analysis of Hit Rate and False Alarm Rate.** In Experiment 1, an ANOVA performed on FARs yielded significant main effects of face race and JOL type, both of which were qualified by a significant interaction. When comparing memory for same-race faces, FARs were significantly lower for those who made delayed, *M* = .16 (.02) than immediate JOLs, *M* = .24 (.02), *t*(189) = 3.23, *p* = .001, 95% CI of the difference [.03, .12], *d* = .45; however, for cross-race faces, FARs were similar for those who made immediate, *M*= .24 (.02), and delayed JOLs, *M* = .22 (.02), *t*(189) = .72, *p* = .47, 95% CI of the difference [-.03, .06], *d* = .13. Subsequent analyses on HR data yielded non-significant main effects of face race and JOL type and a non-significant interaction.

In Experiment 2, an ANOVA performed on FARs yielded a significant main effect of face race; FARs were significantly lower for same-race, *M* = .17 (.01), than cross-race faces, *M* = .22 (.01). An ANOVA performed on HR data yielded significant main effects of face race and JOL type; HRs were significantly higher for same-race, *M* = .61 (.01), than cross-race faces, *M* = .53 (.02), and for those who made delayed, *M* = .60 (.02), than immediate JOLs, *M* = .54 (.02). The interaction was not significant.

**Accuracy of Predictive JOLs**

**Mixed Effects Logistic Regression on Predictive JOLs.** For these analyses of predictive JOLs, we focused on responses to *old* target faces (those for which participants made a JOL), with accuracy coded as 0 = miss and 1 = hit. All participants were included in this analysis. This model consisted of random intercepts of participants (as judgment trials were nested within participants) and face race, JOL type, and JOL level as predictors. JOL level was assessed at six levels of 0, 20, 40, 60, 80, and 100, rather than the aggregated JOL levels for the ANOVAs. Experiment 1 results indicated that JOL level was a significant predictor of recognition accuracy, estimate (log odds) = .14 (.03, z = 4.67, *p* < .001; faces rated with higher JOLs were more likely to be accurately recognized. No other results were significant. Experiment 2 results indicated that JOL level, estimate (log odds) = .25 (.03, z = 7.51, *p* < .001, face race, estimate (log odds) = .31 (.07, z = 4.60, *p* < .001, and JOL type, estimate (log odds) = .35 (.11, z = 3.51, *p* = .002, were all significant predictors of recognition accuracy. Faces rated with higher JOLs, same-race faces, and faces followed by a delayed JOL were more likely to be accurately recognized.

**Analysis of Frequency of High versus Low Predictive JOLs.** To assess whether participants provide high predictive JOL ratings more frequently to same- than cross-race faces, or when making immediate versus delayed JOLs, for each participant, we summed the total number of responses made with a high JOL (80-100) (maximum of 20 total study trials; 10 White and 10 Black) and the total number of responses made with a low JOL (0-20). Then, for each experiment, we conducted two separate 2 (Face Race: White or Black) x 2 (JOL Type: immediate or delay) ANOVAs on the mean number of total responses made at the highest JOL level and at the lowest JOL level. For both analyses, a Bonferroni correction of α = .025 (.05 / # of ANOVAs) was used. All participants were included in these analyses.

Statistics for these analyses are presented in Table S1 in Supplementary Materials. First, analyses of the average number of responses made with a high JOL yielded no significant effects in Experiment 1, but a significant main effect of face race in Experiment 2; participants provided high JOL ratings more often to same-race, *M* = 2.53 (.20), than cross-race faces, *M* = 2.20 (.19). Second, analyses of the average number of responses made with a low JOL yielded a significant main effect of face race in Experiment 1 (but not Experiment 2); participants provided low JOL ratings more often to cross-race, *M* = 3.10 (.21), than same-race faces, *M* = 2.71 (.20). There was also a significant main effect of JOL type in Experiments 1 and 2; participants provided low JOL ratings more often when making delayed [*M_Exp.1_* = 3.47 (.26); *M_Exp.2_* = 2.94 (.27)] than immediate JOLs [*M_Exp.1_* = 2.35 (.27); *M_Exp.2_* = 1.85 (.26)]. These results suggest that participants were metacognitively aware of the relative difficulty remembering cross-race faces and when making delayed JOLs, and this was reflected in the average frequencies of their JOL responses.

**Accuracy of Postdictive Confidence**

**Mixed Effects Logistic Regression on Postdictive Confidence.** For these analyses of postdictive confidence, we focused on “old” responses (similar to analyzing data for “choosers” in an eyewitness identification paradigm), with accuracy coded as 0 = false alarm and 1 = hit. All participants were included in this analysis. Initially, this model consisted of random intercepts of participants (as judgment trials were nested within participants) and face race, JOL type, and confidence as predictors; however, we encountered model convergence issues with this model. Thus, we conducted a fixed effects logistic regression model. Confidence was assessed at the six levels of 0, 20, 40, 60, 80, and 100, rather than the aggregated confidence levels for the ANOVAs.

Experiment 1 results indicated that face race was a significant predictor of accuracy, estimate (log odds) = .20 (.09, z = 1.30, *p* = .02; same-race faces were more likely to be accurate than cross-race faces. JOL type was also a significant predictor of accuracy, estimate (log odds) = .34 (.09, z = 3.85, *p* < .001; participants who had made delayed JOLs were more accurate than participants who had made immediate JOLs. Confidence was also a significant predictor of accuracy, estimate (log odds) = .43 (.03, z = 12.72, *p* < .001; faces rated with higher confidence were more likely to be accurately recognized than faces rated with lower confidence. Experiment 2 results indicated that face race was not a significant predictor of accuracy, estimate (log odds) = .02 (.05, z = .45, *p* = .65. However, replicating Experiment 1, JOL type was a significant predictor of accuracy, estimate (log odds) = .18 (.08, z = 2.18, *p* = .03; participants who had made delayed JOLs were more accurate than participants who had made immediate JOLs. Confidence was also a significant predictor of accuracy, estimate (log odds) = .12 (.02, z = 5.83, *p* < .001; faces rated with higher confidence were more likely to be accurately recognized than faces rated with lower confidence.

**Analysis of Frequency of High versus Low Postdictive Confidence.** To assess whether participants provide high postdictive confidence ratings more frequently to same- than cross-race faces, for each participant, we summed the total number of responses made with confidence ratings of 100 (maximum of 40 total test trials; 20 White and 20 Black) and the total number of responses made with confidence ratings of 0-20. Then, for each experiment, we conducted two separate 2 (Face Race: White or Black) x 2 (JOL Type: immediate or delay) ANOVAs on the mean number of total responses made at the highest postdictive confidence level and the lowest postdictive confidence level. For these analyses, a Bonferroni correction of α = .025 (.05 / # of ANOVAs) was used. All participants were included in these analyses.

Statistics for these ANOVAs are presented in Table S2 in Supplementary Materials. First, a 2 (Face Race: White or Black) x 2 (JOL Type: immediate or delay) ANOVA on the mean number of responses made with confidence ratings of 100 yielded a significant main effect of face race in both Experiments 1 and 2; participants were more likely to make high confidence judgments to same-race [*M_Exp.1_* = 1.93 (.13); *M_Exp.2_* = 5.10 (.32)] than cross-race faces [*M_Exp.1_* = 1.59 (.14); *M_Exp.2_* = 3.88 (.27)]. No other effects were significant.

Second, in Experiment 1, a 2 (Face Race: White or Black) x 2 (JOL Type: immediate or delay) ANOVA on the mean number of responses made with confidence ratings of 0-20 yielded non-significant main effects of face race and JOL type. However, the interaction was significant; for same-race faces, participants in the immediate JOL condition were similarly likely to give low confidence ratings *M* = .79 (.12) as those in delayed JOL condition *M* = .80 (.14), *t*(189) = -.02, *p* = .91, 95% CI of the difference [-.37, .36,], *d < .*001; however, for cross-race faces, participants in the delayed JOL condition were more likely to give low confidence ratings, *M* = .92 (.13), than those in the immediate JOL condition, *M* = .51 (.10), *t*(189) = -2.50, *p* = .01, 95% CI of the difference [-.73, -.09,], *d* = -.36. In Experiment 2, there was a significant main effect of face race; participants were more likely to give low confidence ratings to cross-race *M* = 2.70 (.24) than same-race faces *M* = 2.01 (.20). No other effects were significant.

**Comparing the Proportion Correct based on Different Criteria for “High” Confidence**

To address the potential problem with collapsing predictive and postdictive confidence differently, we computed the proportion correct (1) for high predictive JOLs of 100% only, and (2) for high postdictive confidence ratings collapsed across 80% and 100%. Below, we present the proportion correct data for Experiments 1 and 2 when defining a “high JOL/high confidence” using 80-100% versus 100%-only. The data are presented alongside each other for easy comparison. Overall, the proportion correct for both predictive and postdictive confidence when calculated collapsed across 80-100% were not drastically different than when calculated for 100%-only, with few exceptions. Furthermore, the main conclusion of our paper is not affected by these differences, postdictive confidence is a better indicator of subsequent memory accuracy than predictive confidence.

Experiment 1: Mean Proportion Correct at **High JOLs of 80-100%** (with Standard Error in Parentheses) – As presented in the manuscript

|  | Immediate JOL | Delayed JOL |
| --- | --- | --- |
| Same-Race | .57 (.04) | .71 (.04) |
| Cross-Race | .54 (.04) | .70 (.05) |

Experiment 1: Mean Proportion Correct at **High JOLs of 100% only** (with Standard Error in Parentheses)

|  | Immediate JOL | Delayed JOL |
| --- | --- | --- |
| Same-Race | .46 (.10) | .64 (.10) |
| Cross-Race | .57 (.11) | .69 (.11) |

Experiment 2: Mean Proportion Correct at **High JOLs of 80-100%** (with Standard Error in Parentheses) – As presented in the manuscript

|  | Immediate JOL | Delayed JOL |
| --- | --- | --- |
| Same-Race | .63 (.04) | .78 (.05) |
| Cross-Race | .57 (.05) | .61 (.05) |

Experiment 2: Mean Proportion Correct at **High JOLs of 100%** (with Standard Error in Parentheses)

|  | Immediate JOL | Delayed JOL |
| --- | --- | --- |
| Same-Race | .66 (.08) | .83 (.10) |
| Cross-Race | .63 (.08) | .76 (.10) |

----------------------------------------------------------------------------------------------------------------

Experiment 1: Mean Proportion Correct at **High Confidence of** **80-100%** (with Standard Error in Parentheses)

|  | Immediate JOL | Delayed JOL |
| --- | --- | --- |
| Same-Race | .81 (.02) | .91 (.02) |
| Cross-Race | .84 (.02) | .85 (.02) |

Experiment 1: Mean Proportion Correct at **High Confidence of** **100% only** (with Standard Error in Parentheses) – As presented in the manuscript

|  | Immediate JOL | Delayed JOL |
| --- | --- | --- |
| Same-Race | .85 (.03) | .95 (.03) |
| Cross-Race | .85 (.04) | .90 (.03) |

Experiment 2: Mean Proportion Correct at **High Confidence 80-100%** (with Standard Error in Parentheses)

|  | Immediate JOL | Delayed JOL |
| --- | --- | --- |
| Same-Race | .91 (.02) | .91 (.02) |
| Cross-Race | .89 (.02) | .87 (.02) |

Experiment 2: Mean Proportion Correct at **High Confidence 100% only** (with Standard Error in Parentheses) – As presented in the manuscript

|  | Immediate JOL | Delayed JOL |
| --- | --- | --- |
| Same-Race | .95 (.02) | .93 (.02) |
| Cross-Race | .94 (.03) | .89 (.03) |

Table S1

*Results from Two Separate 2 (Face Race) x 2 (JOL Type, Immediately vs. Delayed) ANOVAs on Average Frequencies of High JOLs and Low JOLs*

|  |  | High JOL | | |  | Low JOL | | |
| --- | --- | --- | --- | --- | --- | --- | --- | --- |
| Exp. | Effect | *F* | *df* |  |  | *F* | *df* |  |
| 1 | Face Race | 0.31 | 1, 189 | .002 |  | 7.12 * | 1, 189 | .04 |
|  | JOL Type | 1.87 |  | 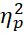 .01 |  | 8.80 ** |  | .04 |
|  | Face Race x JOL Type | 1.42 |  | .01 |  | 0.03 |  | 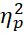< .001 |
|  |  |  |  |  |  |  |  |  |
| 2 | Face Race | 6.25 * | 1, 196 | .03 |  | 1.60 | 1, 196 | .01 |
|  | JOL Type | 0.73 |  | .004 |  | 8.66 ** |  | .04 |
|  | Face Race x JOL Type | 0.02 |  | < .001 |  | 1.42 |  | .01 |

*Note*: df indicates degrees of freedom for each test and effect sizes reported are partial-eta squared. High JOL refers to ratings made with 80-100% confidence; low JOL refers to ratings made with 0-20% confidence.

* *p* < .05 ** *p* < .01 *** *p* < .001

Table S2
 *Results from Two Separate 2 (Face Race) x 2 (JOL Type, Immediate versus Delayed) ANOVAs on Average Frequencies of High and Low Postdictive Confidence*

|  |  | High Postdictive Confidence | | |  | Low Postdictive Confidence | | |
| --- | --- | --- | --- | --- | --- | --- | --- | --- |
| Exp. | Effect | *F* | *df* |  |  | *F* | *df* |  |
| 1 | Face Race | 6.43 * | 1, 189 | .03 |  | 0.94 | 1, 189 | .01 |
|  | JOL Type | 1.03 |  | .01 |  | 1.83 |  | .01 |
|  | Face Race x JOL Type | 0.30 |  | 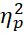.002 |  | 5.85 * |  | 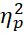.03 |
|  |  |  |  |  |  |  |  |  |
| 2 | Face Race | 42.88 *** | 1, 196 | .18 |  | 25.35 *** | 1, 196 | .12 |
|  | JOL Type | 0.46 |  | .002 |  | 2.61 |  | .01 |
|  | Face Race x JOL Type | 0.32 |  | .002 |  | 1.83 |  | .01 |

*Note:* df indicates degrees of freedom for each test and effect sizes reported are partial-eta squared. High confidence refers to ratings made with 100% postdictive confidence; low confidence refers to ratings made with 0-20% postdictive confidence.

* *p* < .05 ** *p* < .01 *** *p* < .001

Table S3

*Mean Spearman’s Correlations between Predictive JOLs and Postdictive Confidence per Experimental Condition*

|  |  | JOL Type | | | | | |
| --- | --- | --- | --- | --- | --- | --- | --- |
| Exp. | Face Race | | Immediate | |  | Delayed | |
|  |  | | *r*_s_ | *N* |  | *r*_s_ | *N* |
| 1 | Same-Race | | .08 (.06) | 79 |  | .06 (.06) | 81 |
|  | Cross-Race | | .13 (.06) |  |  | .15 (.06) |  |
|  |  | |  |  |  |  |  |
| 2 | Same-Race | | .15 (.05) | 72 |  | .21 (.05) | 70 |
|  | Cross-Race | | .21 (.06) |  |  | .30 (.06) |  |

*Note*: Standard error is reported in parentheses. N indicates sample size per condition.

*Figure S1*. The distribution of the average frequency of responses per JOL level by face race and JOL type in Experiment 1 (top panel) and Experiment 2 (bottom panel). Error bars represent standard error of the mean. Average frequencies were calculated by summing up the total number of trials each participant responded with a particular level of JOL, and then averaging the sum across all participants. The distributions in Experiments 1 and 2 are relatively normally distributed; overall, participants were more likely to provide medium JOLs than low or high JOLs.

*Figure S2*. The distribution of the average frequency of responses per confidence level by face race and JOL type in Experiment 1 (top panel) and Experiment 2 (bottom panel). Error bars represent standard error of the mean. Average frequencies were calculated by summing up the total number of trials each participant responded with a particular level of confidence, and then averaging the sum across all participants. The distributions in Experiments 1 and 2 are slightly negatively skewed; overall, participants were more likely to provide high confidence ratings than low confidence ratings.
